# Interobserver variability of cochlear duct measurements in pediatric cochlear implant candidates

**DOI:** 10.1007/s00405-022-07639-6

**Published:** 2022-09-08

**Authors:** Almir Salkic, Erdem Yildiz, Wolf-Dieter Baumgartner, Lejla Tokic, Sabrina Uscuplic, Senada Sarihodzic, Fuad Brkic, Alen Harcinovic, David T. Liu, Faris F. Brkic

**Affiliations:** 1 Department of Otorhinolaryngology, University Clinical Center Tuzla, Tuzla, Bosnia and Herzegovina; 2 Department of Otorhinolaryngology, Head and Neck Surgery, Medical University of Vienna, Waehringer Guertel 18-20, 1090 Vienna, Austria; 3 Department of Radiology, University Clinical Center Tuzla, Tuzla, Bosnia and Herzegovina

**Keywords:** A value, Cochlear duct length, Cochlear implants, Computer tomography, Pediatric cochlear implantation

## Abstract

**Purpose:**

The objective of the study was to evaluate the proposed cochlear duct length estimation based on the cochlear ‘A value’. Furthermore, we assessed the interobserver variability between radiology and otolaryngology attending physicians and otolaryngology trainees.

**Methods:**

Thirteen pediatric cochlear implant candidates were retrospectively analyzed by three otolaryngology physicians (attending physician, second year, and fourth year trainees) and a radiology attending. The cochlear duct length was calculated based on the formula of Grover et al. The differences in acquired measurements between observers were compared using the Wilcoxon matched signed-rank test.

**Results:**

The differences in measurements between the attending otolaryngologist and radiologist were not statistically different, while several significant differences were observed with regard to measurements of attending doctors compared to both residents. In particular, a significant difference between the second year otolaryngology resident and otolaryngology and radiology attending was observed for one side (right ear *p* = 0.034 and *p* = 0.012, respectively). Moreover, the fourth year resident calculated significantly different cochlear duct measurements when compared to the attending otolaryngologist (left ear *p* = 0.014) and radiologist (right ear *p* = 0.047). Interestingly, differently experienced otolaryngology residents provided significantly different measurements for both ears.

**Conclusions:**

Based on these results, cochlear duct length measurement according to the proposed method may be a reliable and cost-effective method. Indeed, otolaryngology training may be sufficient to provide measurements comparable to radiologists. On the other hand, additional efforts should be invested during otolaryngology training in terms of the evaluation of radiological imaging which may increase the capabilities of otolaryngology residents in this regard.

## Introduction

Cochlear implants (CIs) have been one of the most successful neuroprosthetic devices worldwide [1]. The indication for cochlear implantation is persistent severe to profound sensorineural hearing loss, which has been expanded over the past decades for patients with a residual hearing capacity [2]. Especially early implantation in children at young age is crucial to reduce the effects of the hearing disability [3]. The continuous development of CIs enables the application of improved suitability for the individual patient. For the selection of the ideal CI, size differences in inner ears and the quality of cochlear length measurements play an essential role. Following the first histological study on the variability of cochlear measures in 68 human cochleae in 1938, multiple authors have assessed the cochlear duct measurements in terms of variability [4–8].

The importance of preoperative cochlear length measurements is obvious; the overall cochlear length in humans can vary between 25 and 45 mm in patients [6, 7, 9–11]. Therefore, individually sized electrodes should be chosen to prevent insertion trauma, which might occur in cases of long electrodes [12]. This is, furthermore, highlighted by the fact that deeper parts of the human cochlea are tonotopically organized for speech frequency. Assessing cochlear measurement with computed tomography (CT) scans may prevent using an inadequate electrode, which has been shown to result in poor hearing performance [13–15]. In recent years, there has been a focus on the practical and possibly automatic assessment of cochlear duct length from CT scans [16, 17]. Escudé et al. first described what was later called the ‘A-value’, a measurement of the cochlea which can predict the insertion depth of CIs in patients [18]. The authors defined the A-value as the distance of the round window to the farthest point on the lateral wall of the basal turn within the cochlea. Furthermore, Alexiades et al. showed that this single linear measurement could be used to estimate the cochlear duct length (CDL) [19]. Indeed, in congruence with the cochlear size, CDL varies between individuals [8, 20].

Temporal bone CT and/or magnetic resonance imaging (MRI) scans are the most widely used form of pre-cochlear implantation imaging [21]. Although the accuracy of conducted measurements is worse compared to high scanning quality images, such as micro-CT scans, the CDL prediction with CT remains a standard measurement tool due to its simple and fast acquisition [22, 23]. As medical professionals can conduct the A-value measurements at different career stages, the literature is still sparse on intra- and interobserver variability of these measurements. Some studies in CDL prediction already provide significant differences not only between measurements from different specialists but also between measurement times conducted by the same medical staff [22, 24]. On the other hand, other authors showed high inter-rater reliability in intraclass correlational coefficients and fair to excellent intra-rater reliability in CDL measurements [23, 25]. To the best of our knowledge, the literature remains sparse regarding the pediatric population. Therefore, the aim of the current study was to evaluate the applicability of cochlear duct measurements using the adapted measurement method proposed by Grover et al. [26], which was applied by a specialized radiologist, experienced otolaryngology attending doctor, and physicians in training in a tertiary referral center. Furthermore, the interobserver variability between each measuring physician was assessed.

## Materials and methods

### Patients

The study cohort consisted of 13 pediatric pre-lingually bilateral deaf CI candidates under 4 years. All patients were treated with a single-sided CI after the exclusion of cochlear malformations in the respective temporal bone CT scans. ‘A Values’ of both ear sides in each CT scan were measured retrospectively by a radiologist attending (RAD^a^), an ENT attending (ORL^a^), a second year ENT resident (ORL^b^), and a fourth year ENT resident (ORL^c^), independently. The resulting CDL calculations were performed as previously described by Grover et al. 2018 [26]. In short, the respective inner ear was partially reconstructed, showing the entire basal turn and facilitating the measurement of the largest distance between the round window and lateral wall of the first cochlear turn. All resulting CDLs were calculated with the formula 4.16A–3.98, with A representing the respective ‘A value’ [19].

### Statistics

The statistical analysis was performed with the Statistical Program of Social Sciences (SPSS Version 23.0, IBM Corp., Armonk, NY, USA). The non-normal distribution of the data was assumed based on the histograms. Based on this, descriptive data were presented using median and range. The Wilcoxon matched signed-rank test between individual measurements was performed and p values lower than 0.05 were considered statistically significant.

### Ethical statement

The study approval was received on January 22, 2018, from the ethical committee of the University Clinical Center Tuzla (Approval number: 02-09/2-1/18).

## Results

Seven of 13 pediatric patients (53.8%) were male and the median age at the time of the measurement was 2.9 years (range 1.4–3.9 years). None had any temporal bone pathologies.

Tables 1 and 2 present CDL lengths as measured by each clinician. Summed up, ORL^a^ measured a median CDL of 33.46 mm for both ears (range 29.3–37.62 mm). The RAD^a^ calculated the same CDL only for the left side, and the right ear was measured with a slightly larger CDL of 33.88 mm (range 30.55–37.2 mm). While the CDL results of ORL^c^ were similar, ORL^b^ measured an increased variance (range 29.3–41.78 mm).

**Table 1:** Cochlear duct measurement via preoperative CT scan (measurements in millimeter)

Age, years		ORL^a^		RAD^a^		ORL^b^		ORL^c^	
	Sex	Right ear	Left ear	Right ear	Left ear	Right ear	Left ear	Right ear	Left ear
3.5	m	37.6	37.6	37.6	33.5	33.5	33.5	36.8	36.8
2.7	m	33.5	37.6	37.6	37.6	33.5	33.5	33.0	33.5
2.9	f	37.6	29.3	37.6	37.6	29.3	29.3	33.9	33.9
3.6	m	33.5	33.5	29.3	33.5	29.3	29.3	30.6	30.6
3.4	m	33.5	33.5	33.5	33.5	33.5	33.5	33.0	32.2
1.4	f	29.3	33.5	33.5	33.5	33.5	33.5	33.0	32.6
1.8	f	33.5	33.5	33.5	33.5	29.3	29.3	31.8	32.2
3.2	m	33.5	33.5	33.5	33.5	33.5	33.5	33.5	33.5
3.9	f	33.5	37.6	37.6	33.5	33.5	33.5	33.9	33.5
3.6	f	33.5	33.5	37.6	33.5	33.5	33.5	35.5	34.3
1.6	f	33.5	37.6	37.6	37.6	33.5	33.5	35.5	33.9
1.6	m	37.6	37.6	41.8	37.6	37.6	37.6	37.2	36.4
3.0	m	29.3	33.5	33.5	33.5	33.5	33.5	33.9	33.5

ORL^a^, attending otolaryngology physician

ORL^b^, second year otolaryngology resident

ORL^c^, fourth year otolaryngology resident

RAD^a^ attending radiology physician, measurements in millimeters

**Table 2:** Median, minimum and maximum cochlear duct measurement via preoperative CT scan (measurements in millimeter)

	ORL^a^		ORL^b^		ORL^c^		RAD^a^	
Ear side	Right	Left	Right	Left	Right	Left	Right	Left
Median	33.5	33.5	37.6	33.5	33.45	33.5	33.9	33.5
Minimum	29.3	29.3	29	33.5	29.3	29.3	30.6	30.6
Maximum	37.6	37.6	41.8	37.6	37.6	37.6	37.2	36.8

ORL^a^, attending otolaryngology physician

ORL^b^, second year otolaryngology resident

ORL^c^, fourth year otolaryngology resident

RAD^a^, attending radiology physician, measurements in millimeters

We presented the differences between each measurement as calculated by the Wilcoxon ranked paired test in Table 3. In particular, it revealed no significant difference in CDL measurements between ORL^a^ and Rad^a^ for either ear side (left ear *p* = 0.074; right ear *p* = 0.0937). For the right ear, the Rad^a^ showed significantly different CDL results compared to both residents (*p* = 0.012, *p* = 0.047). However, this was not observed in the left ear (*p* = 0.052, *p* = 0.234). Significant differences were also observed in the comparison of results obtained from ORL^a^ and ORL^b^ (*p* = 0.034), but not in comparison to the results of ORL^c^ (*p* = 0.317). Last, significant differences between CDL measurements of two otolaryngology residents were observed for both ears (left ear *p* = 0.034; right ear *p* = 0.007).

**Table 3:** Wilcoxon matched signed rank test between individual measurements (with shown p values for each comparison)

	ORL^a^	RAD^a^	ORL^b^	ORL^c^
*Right side*				
ORL^a^	x	0.937	**0.034**	0.317
Rad^a^	x	x	**0.012**	**0.047**
ORL^b^	x	x	x	**0.007**
*Left side*				
ORL^a^	x	0.074	0.999	**0.014**
Rad^a^	x	x	0.052	0.234
ORL^b^	x	x	x	**0.034**

ORL^a^, attending otolaryngology physician

ORL^b^, second year otolaryngology resident

ORL^c^, fourth year otolaryngology resident

RAD^a^, attending radiology physician

## Discussion

In the current study, we provided evidence on the suitability of the CDL estimation method as proposed by Grover et al. [26] in pediatric patients. Remarkably, the otolaryngology attending physician provided similar results when compared to the radiologist. However, otolaryngology residents seem to warrant further training and experience to utilize this method for CDL measurement.

Notably, we observed significant differences in measurements performed by otolaryngology and radiology attendings compared to otolaryngology residents. Even among both residents, the measurements were significantly different, further highlighting their need for more training to achieve reproducible results. On the contrary, the long-standing experience of both attendings may be explained by their insignificant difference in CDL results for both inner ear sides of pediatric patients. Thus, the respective measurements might also be accurately performed by an otolaryngologist with finished training without additional quality control via radiology, which could contribute to reduced costs and time of preoperative cochlear implantation planning.

The relatively variable cochlear sizes between individuals require accurate ‘A value’ measurements before CI surgery [20], as the most size adequate implant should be chosen. This way, structural inner ear damage may be prevented, as it may lead to long-term fibrosis and decreased implant function over time. As a result of precise planning via ‘A value’ measurements and further calculations before CI insertion, an improved hearing function should be achieved. This is especially important as the precise intracochlear array positioning ideally covers the functionally deaf parts of the tonotopically organized cochlea without the risk of an intrascalar CI tip dislocation. Most studies with ‘A value’ measurements have been performed on adult patient radiological data. However, it is assumed that the length of the human cochlea is determined by the time of birth, changing skull dimensions and overall growth may have some impact not only on actual inner ear micro-dimensions but also on the overall use of ‘A values’ by professionals due to the rare indication in this patient group. Due to the relatively low number of prelingually deafened patients, the measurement of CDL and ‘A values’ in this patient group is often impossible. Primarily in tertial referral hospitals, it is important to train the staff for these measurements for both adult and pediatric patients. Without this training, it is expected to receive a relatively high interobserver variability in ‘A values’, which can result in poor CI planning.

One crucial factor is that in Bosnia and Herzegovina (and possibly other less developed countries), the implantation of only one CI device insertion in pediatric patients is covered by the general health insurance. Therefore, this highlights the need for even more precise preoperative planning, particularly regarding the electrode length.

The calculated CDL range in this study fits well with the data of previous groups, which confirms a high variability of the cochlear length between individuals [6]. To the best of our knowledge, an interobserver variability study on CDL measurements has never been performed on a pediatric patient population.

The study by Iyaniwura et al. confirmed the variability of CDL measurements obtained from CT scans of cadavers with an average absolute difference of 0.77 ± 0.42 mm [22]. This study revealed a significant difference in ‘A value’ measurements between trained otolaryngology or radiologist specialists. However, this difference was not confirmed in our results. To the best of our knowledge, our study was the first to assess the interobserver variability of CDL calculation between attending otolaryngologists and radiologists and two differently experienced otolaryngology training doctors. The evident differences in measurements between attendings and residents provide valuable viewpoints concerning CDL calculations for pediatric patients. Next to attending radiologists, attending otolaryngology doctors may be suitable for those measurements by providing a low interobserver variability. Regarding the differences in measured results between otolaryngology residents, it might be advisable to emphasize the evaluation of radiological imaging during the otolaryngology residency training schedule. This could ultimately lead to more stable results, better cost-effectiveness, and faster preoperative preparation in pediatric cochlear implantation.

Although this study provided the first insights into CDL measurements between different otolaryngology residents of different training levels, this study also has limitations. First, we included a relatively small cohort of pediatric patients; therefore, multivariate analysis is not applicable. Second, data on interrater reliability and interclass correlation coefficients could not be collected. However, given the fact that significant differences were observed, this study could pave the ground for future work to extend these observations and potentially replicate the results. Indeed, we provided evidence that the proposed method of CDL calculation may be suitable not only for radiologists, but also for experienced otolaryngologists. This may certainly reduce the time and costs of CI planning in pediatric patients.

## Conclusions

The importance of preoperative CDL calculations is emphasized by the significant implications on CI insertion depths considering varying dimensions in pediatric patients. Attending otolaryngologists may be suitable for CDL measurement in a pediatric population prior to CI. This could certainly reduce the costs and time in planning of the CI surgery. On the other hand, our results underline the need for more extensive radiological training for otolaryngology residents to increase their skills in evaluating radiological imaging. Although further studies are needed to validate the current results, we provided insights into the feasibility of ‘A value’ measurement performance by trained professionals.

## Data Availability

Not applicable.
